# Cell culture models for study of differentiated adipose cells

**DOI:** 10.1186/scrt527

**Published:** 2014-12-16

**Authors:** Martin Clynes

**Affiliations:** National Institute for Cellular Biotechnology, Dublin City University, Glasnevin, Dublin 9, Ireland

## Abstract

Adipose cells are an important source of mesenchymal stem cells and are important for direct use in research on lipid metabolism and obesity. In addition to use of primary cultures, there is increasing interest in other sources of larger numbers of cells, using approaches including induced pluripotent stem cell differentiation and viral immortalisation.

There is increasing interest in the use of adipose cells, both brown and white types, not least because the obesity epidemic dictates a need for increased research on adipose tissue and lipid metabolism. The paper by Balducci and colleagues – a collaboration between five Italian groups – reports the immortalisation, by lentiviral transduction, of human adipose-derived stromal cells [1].

Adipose cells are an important resource for biomedical research, partly because of the ready availability of human surgical material and the fact that they can be used to generate mesenchymal stem cells [2] – which themselves have interesting differentiation potential, for example towards a hepatocyte phenotype [3] – and even pluripotent stem cells [4]. Adipose-derived stem cells have been reported to differentiate into osteoblasts, chondrocytes, myocytes and neurons, as well as back to adipocytes, depending on the culture conditions [5]. Pluripotent stem cells [6, 7], including induced pluripotent stem cells [8], can be differentiated *in vitro* into cells with multiple phenotypic characteristics of adipose cells, including specifically brown adipose cells [7].

It is interesting to note that telomerase expression in bone marrow stromal cells resulted in enhanced bone formation [9, 10]. Another approach to generating large populations of adipose cells *in vitro* is to use viral immortalisation, as has been also achieved in other systems such as bone marrow progenitor cells [11].

Balducci and colleagues report diversity in differentiation potential between cell lines immortalised with different gene combinations, which is in itself an interesting observation, but it is not entirely clear what the cellular or molecular basis for this may be or whether it is a purely random observation [1]. Whatever the mechanism, human adipose-derived stromal cells co-transduced with human telomerase reverse transcriptase and human papilloma virus E6/E7 generated immortalised cells that retained the capacity to differentiate down osteogenic and adipogenic lineages and to produce angiogenesis-related proteins. Cells transduced with human telomerase reverse transcriptase alone or with human telomerase reverse transcriptase and Simian virus 40 did not retain these capacities to the same extent. The availability of immortal cell lines that closely resemble adipose-derived stromal cells contributes a useful new resource for those working on this fascinating cell type.

Nevertheless, it is important to bear in mind that, as in all such cases, these adipose cells are not normal cells identical to their parental finite-lifespan progenitors – transduced cells are unlikely to be acceptable for therapeutic use except perhaps in the terminal stages of life-threatening diseases. Indeed, Balducci and colleagues acknowledge this limitation and report on chromosomal aberrations and unbalanced translocations in the transduced cells. However, there is no doubt that the availability of these immortalised human adipose-derived stromal cell lines will significantly facilitate research on this interesting and relatively neglected cell type, and availability of these cell lines will help to answer more rapidly questions about their biology and expedite their application in cell therapy/tissue engineering, even if the cells eventually used for therapy will most probably be of primary origin rather than cell lines. The availability of large numbers of these cells that can be easily grown also offers the potential for discovery of additional autocrine, paracrine and endocrine factors which these cells may produce, but at levels too low to be detected from small-scale, limited-lifespan primary cultures, and this, in the end, could be the most valuable legacy from this interesting paper.

The availability of these different sources of adipose cells provides a much-expanded toolkit for research on adipose cells *in vitro*, and should make a significant impact on the progress of obesity research and on our understanding of adipose cell differentiation.
